# Deep learning for automatic volumetric bowel segmentation on body CT images

**DOI:** 10.1007/s00330-025-11623-z

**Published:** 2025-05-02

**Authors:** Junghoan Park, Sungeun Park, Han-Jae Chung, Da In Lee, Jong-min Kim, Se Hyung Kim, Eun Kyung Choe, Kyu Joo Park, Soon Ho Yoon

**Affiliations:** 1 Department of Radiology, Seoul National University Hospital, Seoul National University College of Medicine, Seoul, Republic of Korea; 2 Department of Radiology, Konkuk University Medical Center, Konkuk University School of Medicine, Seoul, Republic of Korea; 3 AI Center, MEDICAL IP Co., Ltd., Seoul, Republic of Korea; 4 Institute of Radiation Medicine, Seoul National University Medical Research Center, Seoul, Republic of Korea; 5 Healthcare Research Institute, Seoul National University Hospital Healthcare System Gangnam Center, Seoul, Republic of Korea; 6 Department of Surgery, Seoul National University College of Medicine, Seoul, Republic of Korea

**Keywords:** Deep learning, Tomography (X-ray computed), Gastrointestinal tract, Constipation

## Abstract

**Objectives:**

To develop a deep neural network for automatic bowel segmentation and assess its applicability for estimating large bowel length (LBL) in individuals with constipation.

**Materials and methods:**

We utilized contrast-enhanced and non-enhanced abdominal, chest, and whole-body CT images for model development. External testing involved paired pre- and post-contrast abdominal CT images from another hospital. We developed 3D nnU-Net models to segment the gastrointestinal tract and separate it into the esophagus, stomach, small bowel, and large bowel. Segmentation accuracy was evaluated using the Dice similarity coefficient (DSC) based on radiologists’ segmentation. We employed the network to estimate LBL in individuals having abdominal CT for health check-ups, and the height-corrected LBL was compared between groups with and without constipation.

**Results:**

One hundred thirty-three CT scans (88 patients; age, 63.6 ± 10.6 years; 39 men) were used for model development, and 60 for external testing (30 patients; age, 48.9 ± 15.8 years; 16 men). In the external dataset, the mean DSC for the entire gastrointestinal tract was 0.985 ± 0.008. The mean DSCs for four-part separation exceeded 0.95, outperforming TotalSegmentator, except for the esophagus (DSC, 0.807 ± 0.173). For LBL measurements, 100 CT scans from 51 patients were used (age, 67.0 ± 6.9 years; 59 scans from men; 59 with constipation). The height-corrected LBL were significantly longer in the constipation group on both per-exam (79.1 ± 12.4 vs 88.8 ± 15.8 cm/m, *p* = 0.001) and per-subject basis (77.6 ± 13.6 vs 86.9 ± 17.1 cm/m, *p* = 0.04).

**Conclusion:**

Our model accurately segmented the entire gastrointestinal tract and its major compartments from CT scans and enabled the noninvasive estimation of LBL in individuals with constipation.

**Key Points:**

***Questions*** *Automated bowel segmentation is a first step for algorithms, including bowel tracing and length measurement, but the complexity of the gastrointestinal tract limits its accuracy*.

***Findings*** *Our 3D nnU-Net model showed high performance in segmentation and four-part separation of the GI tract (DSC > 0.95), except for the esophagus*.

***Clinical relevance*** *Our model accurately segments the gastrointestinal tract and separates it into major compartments. Our model potentially has use in various clinical applications, including semi-automated measurement of LBL in individuals with constipation*.

**Graphical Abstract:**

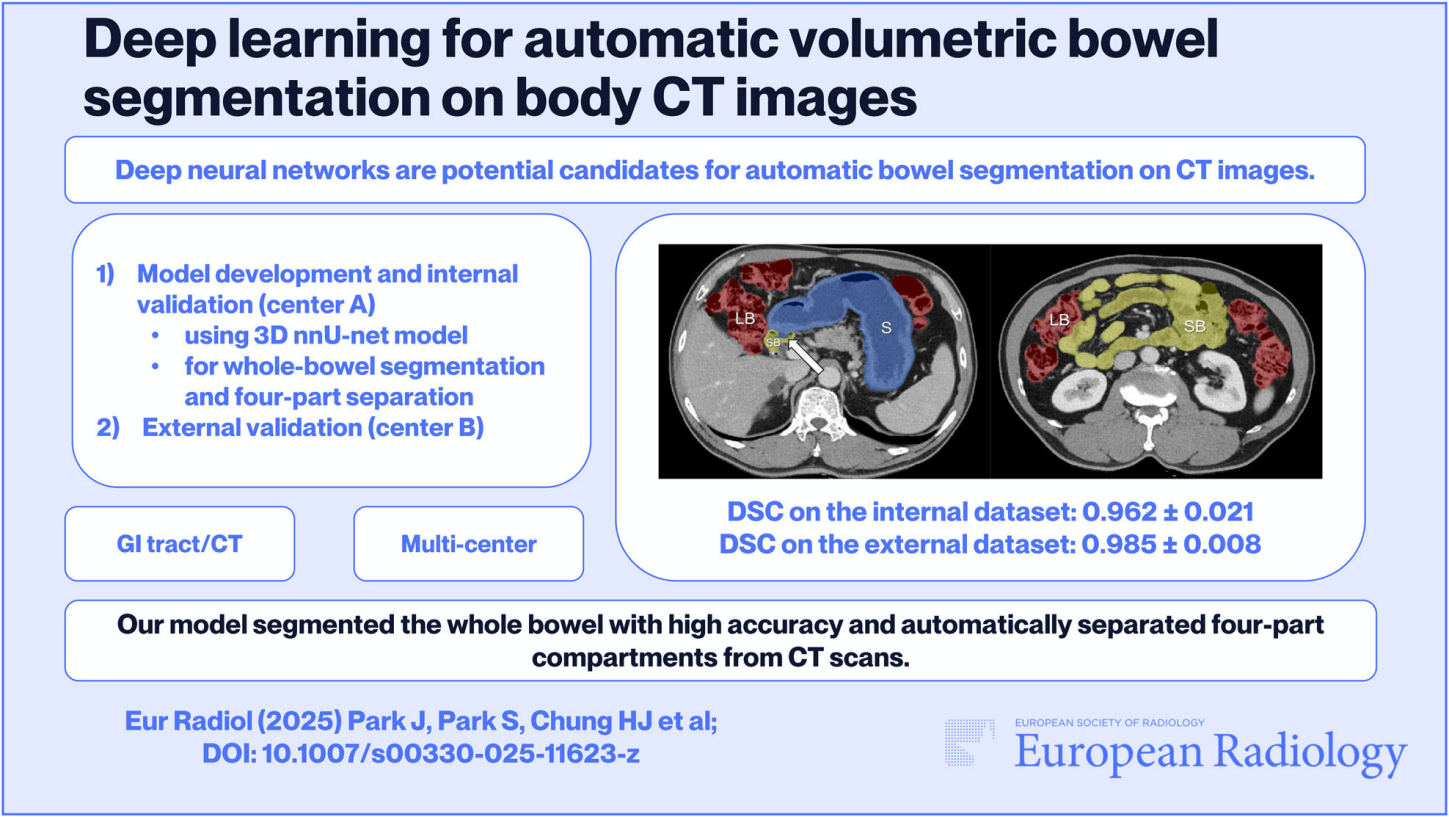

## Introduction

Abdominal CT is the modality of choice for diagnosing various bowel diseases, allowing the assessment and localization of inflammation, obstruction, or perforation by tracing the bowel loop [1, 2]. It is particularly crucial for evaluating the small bowel, which is not easily accessible via endoscopy [3]. Tracing the gastrointestinal (GI) tract is tedious and can be challenging, especially when the bowel is not distended. Moreover, the GI tract varies in length and course among individuals and is movable within the mesentery, complicating the tracing process even for the same patient across different CT scans. Consequently, radiologists—especially those with less experience—often spend considerable time on bowel assessment and may sometimes make incorrect diagnoses due to time constraints [4, 5].

Bowel length also has important implications in GI diseases. For example, patients with Crohn’s disease or necrotizing enterocolitis sometimes need recurrent bowel resection for treatment [6, 7]. For these patients, estimating the postoperative residual length of the small bowel is critical because a short remaining small bowel can result in malnutrition, known as short bowel syndrome [8]. In addition, reports have suggested an association between the length of the entire colon or rectosigmoid colon and constipation [9, 10]. Cross-sectional imaging may serve as a noninvasive tool for measuring bowel length, but imaging-based measurements are challenging due to their time-consuming and labor-intensive nature, as well as the difficulty in consistently tracing the bowel loop. A study reported semi-automatic measurement of the small bowel length using MR enterography, but it took over 30 min per case, making it impractical for clinical use [11].

Automated algorithms for tracing the bowel, detecting diseased segments, and measuring bowel length would help overcome these challenges. Segmenting the bowel from CT images is the first step in such algorithms, and deep neural networks are potential candidates [12, 13]. However, deep neural networks for GI tract segmentation suffer from limited accuracy [14, 15], and the inherent complexity of the GI tract hinders their development. This study aimed to develop a fully capable deep neural network for automatic bowel segmentation on CT images and to evaluate its applicability for estimating large bowel length (LBL) in patients with constipation.

## Materials and methods

The institutional review boards of participating hospitals approved this retrospective study (IRB No. 2207-038-1337 from center A/C, 2022-07-023 from center B), which aimed to develop a deep neural network for automatic bowel segmentation, and the requirement for informed consent was waived due to its retrospective nature. Center C is a subsidiary hospital of Center A, and they share the same IRB approval. Making the model publicly available is not currently feasible due to commercialization considerations.

### Study population

**Fig. 1:**
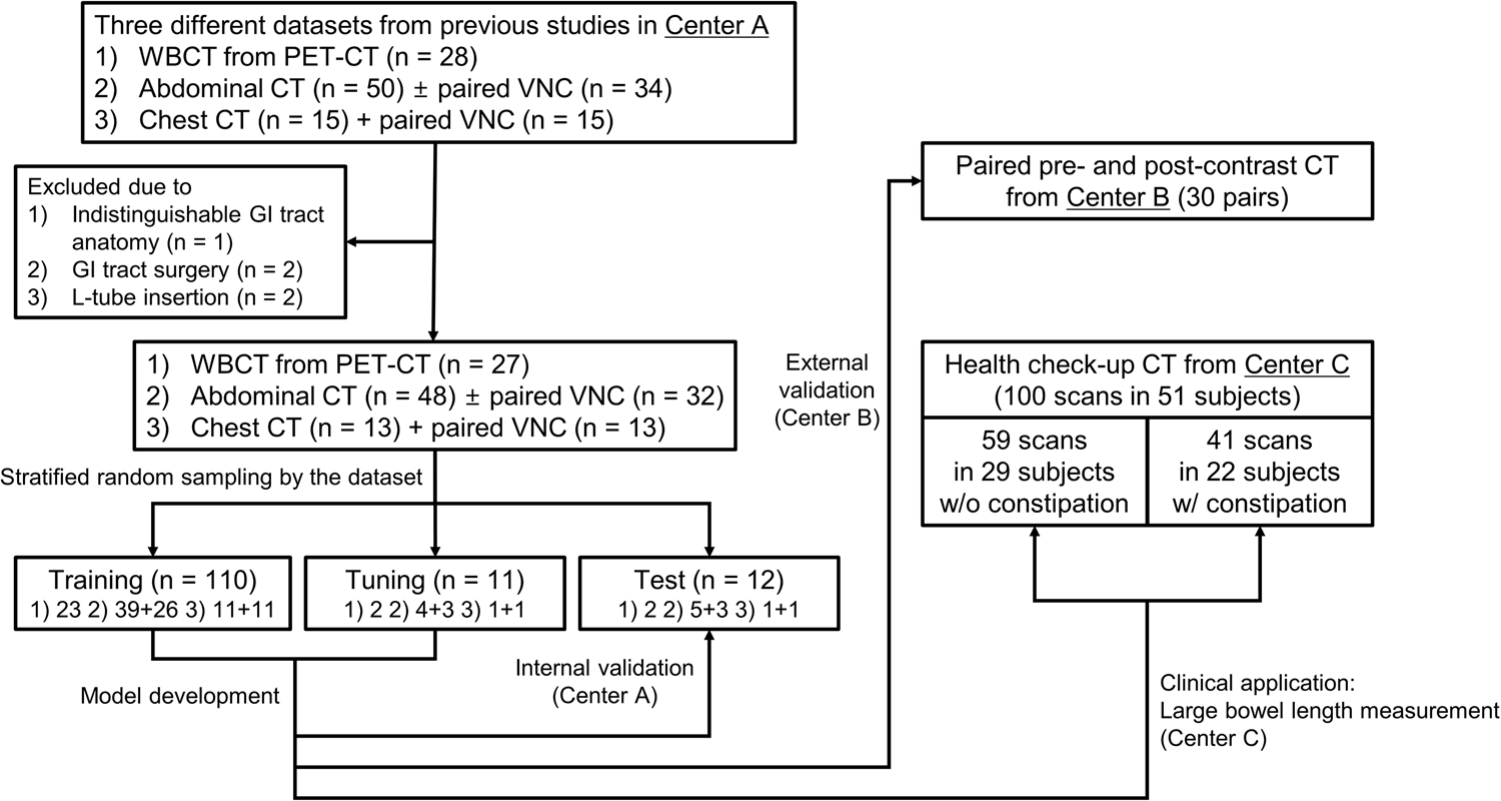
Study diagram. GI, gastrointestinal; VNC, virtual noncontrast image; WBCT, whole-body CT

For model development, we used various non-enhanced and contrast-enhanced body CT scans from a single tertiary hospital (Center A): (1) contrast-enhanced abdominal CT with or without paired virtual non-contrast CT (VNC) images, (2) contrast-enhanced chest CT with paired VNC images, and (3) non-enhanced whole-body CT (WBCT) as part of whole-body PET-CT scans (Fig. 1). All VNC images were clinically obtained using commercially available post-processing systems implemented in the vendors’ platforms (i.e., Syngo.via for the SOMATOM Force [Siemens Healthineers] and IntelliSpace Portal for IQon Spectral CT [Philips Healthcare]). All these scans included portions of the GI tract but exhibited varying anatomic coverage. Rather than selecting an optimal input, we intentionally incorporated this variability to ensure robust segmentation across diverse body regions, imaging protocols, and irrespective of contrast enhancement. All dataset used in this study were randomly selected from a portion of the datasets that were previously used in other studies: a total of 50 abdominal CT exams out of 125, 15 chest CT exams out of 104, and 28 WBCT exams out of 100 were randomly selected for this study (Supplemental Text) [16–18]. The prior studies dealt with segmenting the liver, pulmonary vessels, or body composition, whereas in this manuscript, we segmented bowel structures [16–18]. Scans were excluded if (1) any GI part except the appendix had been surgically resected (*n* = 2), (2) intervention-related foreign bodies were inserted in the GI tract (*n* = 2), or (3) the GI tract was indistinguishable because of severe artifacts or pathologies (*n* = 1). Finally, a total of 133 CT scans—48 abdominal CT exams (32 with paired VNC images), 13 chest CT exams with paired VNC images, and 27 WBCT exams—from 88 patients were included.

For external testing, we included patients over 18 years who underwent pre- and post-contrast abdominal CT scans as part of a single abdominal CT protocol for clinical purposes at another tertiary hospital (Center B) between September and December 2021. The same exclusion criteria were applied, and 30 pre- and post-contrast pairs were included. The sample size was estimated to be 63, assuming a target Dice Similarity Coefficient (DSC) of 0.95 for our model and a standard deviation of 0.05 for the DSC difference between our model and TotalSegmentator v2.2.1, based on the reported DSC of 0.932 for TotalSegmentator v2.2.1 on external datasets [19]. However, due to practical constraints in the workforce and the time required for creating ground-truth masks, we limited the number of cases to 30 pairs (60 scans in total) instead of 63 pairs.

For estimating LBL, we enrolled 51 randomly selected subjects who visited the other tertiary hospital (Center C) for a health check-up, including abdominal CT and anoscopy between 2003 and 2022. Participants underwent a detailed interview regarding their bowel habits and were evaluated for anatomical abnormalities through digital rectal examination and anoscopy. The constipation (*n* = 29) and normal bowel habit groups (*n* = 22) were distinguished based on the Rome IV criteria, assessing factors such as straining, stool consistency, sensation of incomplete evacuation/anorectal obstruction, the necessity of manual maneuvers for defecation, small bowel movement frequency, and use of laxatives (Fig. 1) [20].

### Data preparation

All CT images were stored as anonymized DICOM files before being processed using commercially available software (MEDIP v.2.3.0.4410., MEDICAL IP) to create ground-truth masks of the GI tract. The ground-truth masks were semi-automatically generated to enhance efficiency and reduce processing time, as follows. First, the internal organ masks were automatically generated by the software using a commercially available model [18]. Next, preliminary masks of the whole GI tract were automatically created by removing the masks of the abdominal solid organs (liver, gallbladder, pancreas, spleen, both kidneys, and urinary bladder), heart, and major vessels from the internal organ masks, with these organ masks being generated using previously trained networks. Then, technicians roughly refined the preliminary masks, which were subsequently reviewed and manually refined slice-by-slice by one of two board-certified radiologists (both with 7 years of experience in body CT interpretation) to create the ground-truth masks. Finally, the radiologists cropped the ground-truth masks of the whole GI tract into four parts: esophagus, stomach, small bowel, and large bowel. The ground-truth masks were drawn from the thoracic inlet to the anorectal angle, including both the wall and lumen of the GI tract. For four-part separation of the ground-truth masks, each part of the GI tract was defined as follows: (1) esophagus, from the thoracic inlet to the gastroesophageal junction, (2) stomach, from the gastroesophageal junction to the pylorus, (3) small bowel, from the duodenal bulb to the ileocecal valve, and (4) large bowel, from the cecum to the anorectal angle including appendix (if not surgically resected). When CT scans did not cover the entire GI tract (e.g., esophagus on abdominal CT and stomach, small bowel, and large bowel on chest CT), every portion of the covered GI tract was included in the masks.

### Development and validation of a neural network

Our nnU-net-based network development involved two primary tasks: (1) segmentation of the entire GI tract in CT images and (2) four-part separation of the segmented GI tract into the esophagus, stomach, small bowel, and large bowel [21]. The first network received the internal organ area (predicted using a body component segmentation algorithm from previous research) as input data and produced the whole GI tract area as output data [18]. The second network received the whole GI tract area as input data and produced the esophagus, stomach, small bowel, and large bowel areas as output data. During development, the ground-truth masks of the whole GI tract were used as input data for the second network. However, for internal and external testing, we used the first network’s output data as the input for the second network to perform whole GI tract segmentation and four-part separation simultaneously as a one-step process, assuming the absence of ground-truth masks of the whole GI tract for the four-part separation. This approach was employed to evaluate the accuracy of our network in a real clinical setting.

The development dataset was randomly divided into training, tuning, and internal test sets with a ratio of roughly 8:1:1 (110 CT scans for training, 11 for tuning, and 12 for internal testing), stratified by the CT protocol (Fig. 1). The dataset split was done at the patient level. However, the contrast-enhanced and VNC images were used independently as inputs, even for paired contrast-enhanced and VNC images from the same patient. The nnU-Net can adapt preprocessing strategies and network hyperparameters according to the dataset (e.g., patch size, number of pooling layers, or convolutional kernel size). The patch size was initialized to the median image shape and iteratively reduced while adapting the network topology accordingly until the network could be trained with a batch size of at least two given graphic processing unit memory constraints. The final patch size configurations were 80 × 160 × 160 for the first network and 64 × 160 × 160 for the second network.

**Fig. 2:**
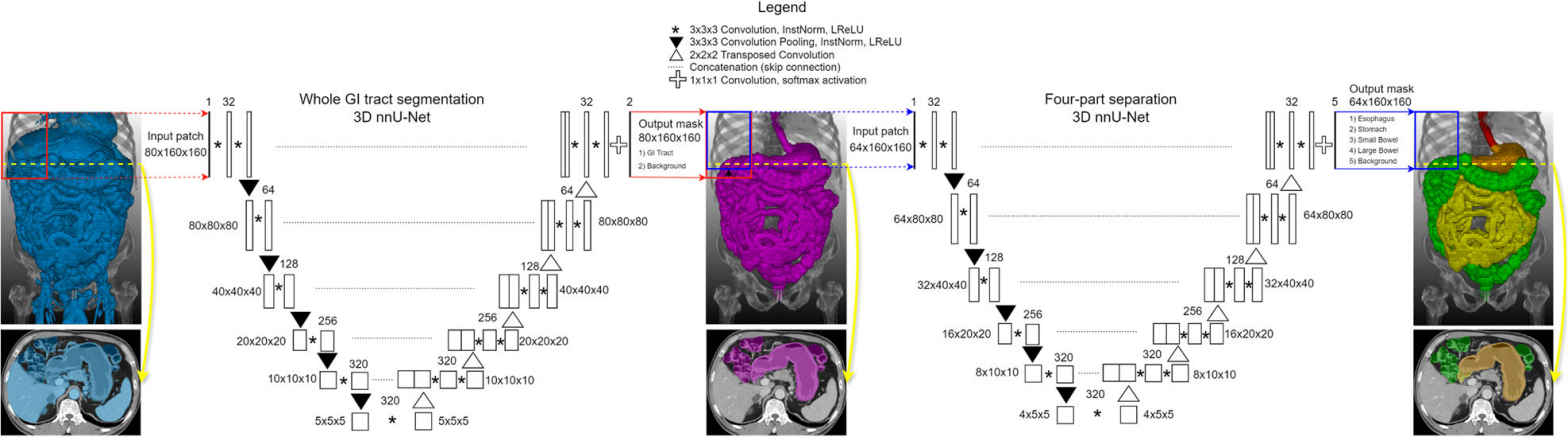
The architecture of our model uses a two-step segmentation approach: whole GI tract segmentation (left), followed by four-part separation (right). GI, gastrointestinal; InstNorm, instance normalization; LReLU, leaky rectified linear unit

3D nnU-Net model parameters were initialized using the He Initialization method. Network optimization was performed using the stochastic gradient descent algorithm and a combination of the cross-entropy loss and the dice loss functions after the final softmax layer. The polynomial learning rate scheduler was initialized at 0.01. Before training, soft and sharp kernel conversion was applied as data augmentation to increase the variety of training data. During training, on-the-fly data augmentation methods such as mirroring, scaling, and rotation were randomly applied. The final model was selected based on the highest average Dice similarity across the tuning dataset. PyTorch 1.11.0 and NVIDIA GeForce RTX 3090 were used during the training and validation process (Fig. 2).

### Comparing LBL according to constipation

Since some patients underwent multiple CT scans, a total of 100 CT scans from 51 patients (59 scans from 29 patients with constipation and 41 scans from 22 patients with normal bowel habits) were used for the LBL analysis. LBL was estimated using all CT scans through the following methods, assuming that the large bowel is a long, cylindrical structure. This assumption allowed us to estimate LBL by dividing the volume by the cross-sectional area of the cylinder.

First, our model automatically segmented the large bowel and calculated its volume. Next, a board-certified abdominal radiologist (J.P.) measured the diameter in the ascending, transverse, descending, and sigmoid colon, respectively, at positions where there was neither excessive dilatation nor collapse. Based on these measurements, the estimated cross-sectional area of the four segments was calculated using the formula π × (diameter/2)^2^. The average of these areas was determined as the average cross-sectional area of the large bowel. Finally, model-calculated LBL was estimated by dividing the volume of the large bowel, calculated by our model, by the estimated average cross-sectional area. The height-corrected length was calculated by dividing the LBL by height or height squared. For comparison purposes, the LBL and height-corrected length were calculated in the same manner using the semi-automatically segmented large bowel mask by a radiologist (i.e., human-generated LBL) for 20 scans from 20 patients (10 from the normal bowel habit group and 10 from the constipation group).

### Statistical analysis

To evaluate segmentation accuracy for the whole bowel and four bowel segments, the DSC, sensitivity, and precision were calculated between ground-truth and network-derived masks (Supplemental Text). Poor segmentation was defined as a DSC < 0.80 [22]. Segmentation accuracy between the internal and external datasets was compared using either the *t*-test or Mann–Whitney *U*-test, depending on the normality, and the external dataset was divided into pre- and post-contrast datasets to compare the segmentation accuracy between the CT scans using the paired *t*-test. Additionally, the segmentation accuracy between our network and TotalSegmentator v2.2.1 or preliminary masks created by MEDIP software was also compared using the paired *t*-test or Wilcoxon signed-rank test, depending on the normality [19] (Supplemental Text). LBL was compared between patients with and without constipation using the independent *t*-test. The correlation between the model-calculated and human-generated LBL was assessed using the intraclass correlation coefficient. A *p*-value < 0.05 was considered statistically significant. Statistical analysis was performed using R statistical software (version 4.2.0; R Foundation for Statistical Computing).

## Results

### Segmentation performance

The mean age and sex distribution of the subjects in the development and external datasets were 63.6 ± 10.6 years (women, 55.6%) and 48.9 ± 15.8 years (women, 46.7%), respectively (Table 1). Our 3D nnU-Net-based segmentation model achieved accurate whole GI tract segmentation in both the internal and external datasets. For the internal dataset, the average DSC, sensitivity, and precision values were 0.962 ± 0.021, 0.970 ± 0.011, and 0.954 ± 0.033, respectively (Table 2). For the external dataset, the accuracy of whole GI tract segmentation was even higher, with the average DSC, sensitivity, and precision being 0.985 ± 0.008, 0.985 ± 0.011, and 0.984 ± 0.007, respectively (Table 3). The external dataset showed significantly higher DSC, sensitivity, and precision compared to the internal dataset (*p* ≤ 0.001) (Supplementary Table 1). In contrast, the preliminary masks showed DSC, sensitivity, and precision values of 0.730 ± 0.157, 0.973 ± 0.017, and 0.605 ± 0.170, respectively, in the internal dataset, and 0.816 ± 0.036, 0.996 ± 0.007, and 0.693 ± 0.052, respectively, in the external dataset. The DSC and precision of our model were substantially higher (*p* < 0.001). The sensitivity of the preliminary masks was statistically significantly higher in the external dataset (*p* < 0.001), but the difference was small (Supplementary Table 2 and Supplementary Fig. 1). No serious errors in whole GI tract segmentation occurred in the external dataset: The DSCs were more than 0.95 in all cases. There was no significant difference in DSC (*p* = 0.16) and sensitivity (*p* = 0.68) between pre- and post-contrast datasets. Precision was significantly lower in the post-contrast dataset, but the difference was small (0.986 ± 0.006 vs 0.983 ± 0.007, *p* < 0.001) (Table 3).

**Table 1:** Baseline characteristics of the subjects enrolled for model development, external validation, and LBL measurement

	Model development	External validation	LBL measurement, per-subject basis^a^			LBL measurement, per-exam basis		
	All	All	No constipation	Constipation	*p*-value	No constipation	Constipation	*p*-value
Number of exams	88 (+45 paired VNC)	30 (pre-/post-contrast pairs)	22	29	–	41	59	–
Age (years)	63.6 ± 10.6	48.9 ± 15.8	68.5 ± 6.3	68.2 ± 9.2	0.55	67.6 ± 6.2	66.6 ± 7.3	0.64
Female sex	49 (55.6%)	14 (46.7%)	5 (22.7%)	15 (51.7%)	0.07	9 (22.0%)	32 (54.2%)	0.003
Height (cm)	N/A	N/A	165.4 ± 9.0	162.0 ± 8.7	0.18	165.7 ± 9.3	161.7 ± 7.6	0.02
CT protocol	48 CECT (+32 paired VNC) 13 PCTA (+13 paired VNC) 27 WBCT	30 paired NECT/CECT	22 CECT	28 CECT 1 CTC	1.00	1 NECT 40 CECT	53 CECT 6 CTC	0.03

*CECT* contrast-enhanced abdominal CT, *CTC* CT colonography, *LBL* large bowel length, *N/A* not applicable, *NECT* non-enhanced abdominal CT, *PCTA* pulmonary CT angiography, *WBCT* non-enhanced whole-body CT

^a^ For subjects who underwent more than one CT examination, the latest CT scan was used for the analysis

**Table 2:** The performance of our model and TotalSegmentator 2.2.1 for the segmentation of the GI tract in the internal dataset

	Our model			TotalSegmentator v2.2.1			*p*-value^a^		
	DSC	Sensitivity	Precision	DSC	Sensitivity	Precision	DSC	Sensitivity	Precision
Whole GI tract segmentation	0.962 ± 0.021	0.970 ± 0.011	0.954 ± 0.033	0.804 ± 0.138	0.788 ± 0.094	0.832 ± 0.180	0.001	< 0.001	0.02
Four-part separation									
Esophagus	0.821 ± 0.044	0.829 ± 0.063	0.826 ± 0.099	0.716 ± 0.040	0.691 ± 0.087	0.760 ± 0.098	< 0.001	< 0.001	0.002
Stomach	0.944 ± 0.036	0.962 ± 0.016	0.929 ± 0.061	0.629 ± 0.170	0.581 ± 0.118	0.705 ± 0.219	< 0.001	< 0.001	0.005
Small bowel	0.934 ± 0.025	0.931 ± 0.034	0.938 ± 0.024	0.754 ± 0.101	0.745 ± 0.174	0.798 ± 0.075	< 0.001	0.002	< 0.001
Large bowel	0.953 ± 0.018	0.963 ± 0.016	0.943 ± 0.030	0.811 ± 0.051	0.797 ± 0.041	0.834 ± 0.100	< 0.001	< 0.001	0.002

Data are presented with mean ± standard deviation

*DSC* Dice similarity coefficient, *GI* gastrointestinal

^a^ *p*-value for the difference of each statistical parameter between our model and TotalSegmentor 2.2.1

**Table 3:** The performance of our model and TotalSegmentator 2.2.1 for the segmentation of the GI tract in the external dataset

	Our model			TotalSegmentator v2.2.1			*p*-value^a^		
	DSC	Sensitivity	Precision	DSC	Sensitivity	Precision	DSC	Sensitivity	Precision
Whole dataset (30 pre-/post-contrast pairs)									
Whole GI tract segmentation	0.985 ± 0.008	0.985 ± 0.011	0.984 ± 0.007	0.846 ± 0.022	0.827 ± 0.050	0.870 ± 0.043	< 0.001	< 0.001	< 0.001
Four-part separation									
Esophagus	0.807 ± 0.173	0.784 ± 0.224	0.877 ± 0.066	0.764 ± 0.052	0.799 ± 0.071	0.742 ± 0.085	0.07	0.66	< 0.001
Stomach	0.970 ± 0.047	0.968 ± 0.074	0.975 ± 0.020	0.597 ± 0.129	0.603 ± 0.135	0.620 ± 0.176	< 0.001	< 0.001	< 0.001
Small bowel	0.960 ± 0.029	0.963 ± 0.031	0.958 ± 0.035	0.820 ± 0.055	0.805 ± 0.101	0.844 ± 0.037	< 0.001	< 0.001	< 0.001
Large bowel	0.963 ± 0.024	0.964 ± 0.028	0.962 ± 0.031	0.853 ± 0.047	0.826 ± 0.050	0.886 ± 0.063	< 0.001	< 0.001	< 0.001
Pre-contrast (*n* = 30)									
Whole GI tract segmentation	0.986 ± 0.007	0.985 ± 0.010	0.986 ± 0.006	0.848 ± 0.022	0.832 ± 0.050	0.868 ± 0.043	< 0.001	< 0.001	< 0.001
Four-part separation									
Esophagus	0.740 ± 0.215	0.681 ± 0.258	0.883 ± 0.073	0.748 ± 0.055	0.807 ± 0.082	0.706 ± 0.079	0.85	0.03	< 0.001
Stomach	0.971 ± 0.043	0.967 ± 0.069	0.977 ± 0.012	0.605 ± 0.107	0.599 ± 0.112	0.638 ± 0.152	< 0.001	< 0.001	< 0.001
Small bowel	0.963 ± 0.025	0.966 ± 0.025	0.960 ± 0.030	0.827 ± 0.050	0.822 ± 0.095	0.841 ± 0.037	< 0.001	< 0.001	< 0.001
Large bowel	0.965 ± 0.022	0.964 ± 0.028	0.967 ± 0.027	0.858 ± 0.043	0.831 ± 0.052	0.889 ± 0.054	< 0.001	< 0.001	< 0.001
Post-contrast (*n* = 30)									
Whole GI tract segmentation	0.984 ± 0.009	0.986 ± 0.012	0.983 ± 0.007	0.844 ± 0.023	0.822 ± 0.050	0.871 ± 0.044	< 0.001	< 0.001	< 0.001
Four-part separation									
Esophagus	0.874 ± 0.074	0.887 ± 0.116	0.870 ± 0.057	0.781 ± 0.044	0.791 ± 0.058	0.778 ± 0.076	< 0.001	0.001	< 0.001
Stomach	0.968 ± 0.051	0.969 ± 0.079	0.972 ± 0.026	0.589 ± 0.149	0.606 ± 0.156	0.602 ± 0.197	< 0.001	< 0.001	< 0.001
Small bowel	0.957 ± 0.033	0.959 ± 0.037	0.956 ± 0.040	0.812 ± 0.060	0.788 ± 0.105	0.848 ± 0.038	< 0.001	< 0.001	< 0.001
Large bowel	0.961 ± 0.027	0.965 ± 0.028	0.958 ± 0.034	0.849 ± 0.050	0.820 ± 0.049	0.882 ± 0.071	< 0.001	< 0.001	< 0.001
*p*-value between pre- and post-contrast^b^									
Whole GI tract segmentation	0.16	0.68	< 0.001	0.053	< 0.001	0.30			
Four-part separation									
Esophagus	< 0.001	< 0.001	0.31	0.006	0.10	< 0.001			
Stomach	0.31	0.63	0.20	0.31	0.65	0.09			
Small bowel	0.055	0.18	0.21	< 0.001	< 0.001	0.13			
Large bowel	0.14	0.83	0.01	0.01	0.009	0.22			

Data are presented with mean ± standard deviation

*DSC* Dice similarity coefficient, *GI* gastrointestinal

^a^ *p*-value for the difference of each statistical parameter between our model and TotalSegmentor 2.2.1

^b^ *p*-value for the difference of each statistical parameter between pre-contrast and post-contrast datasets

**Fig. 3:**
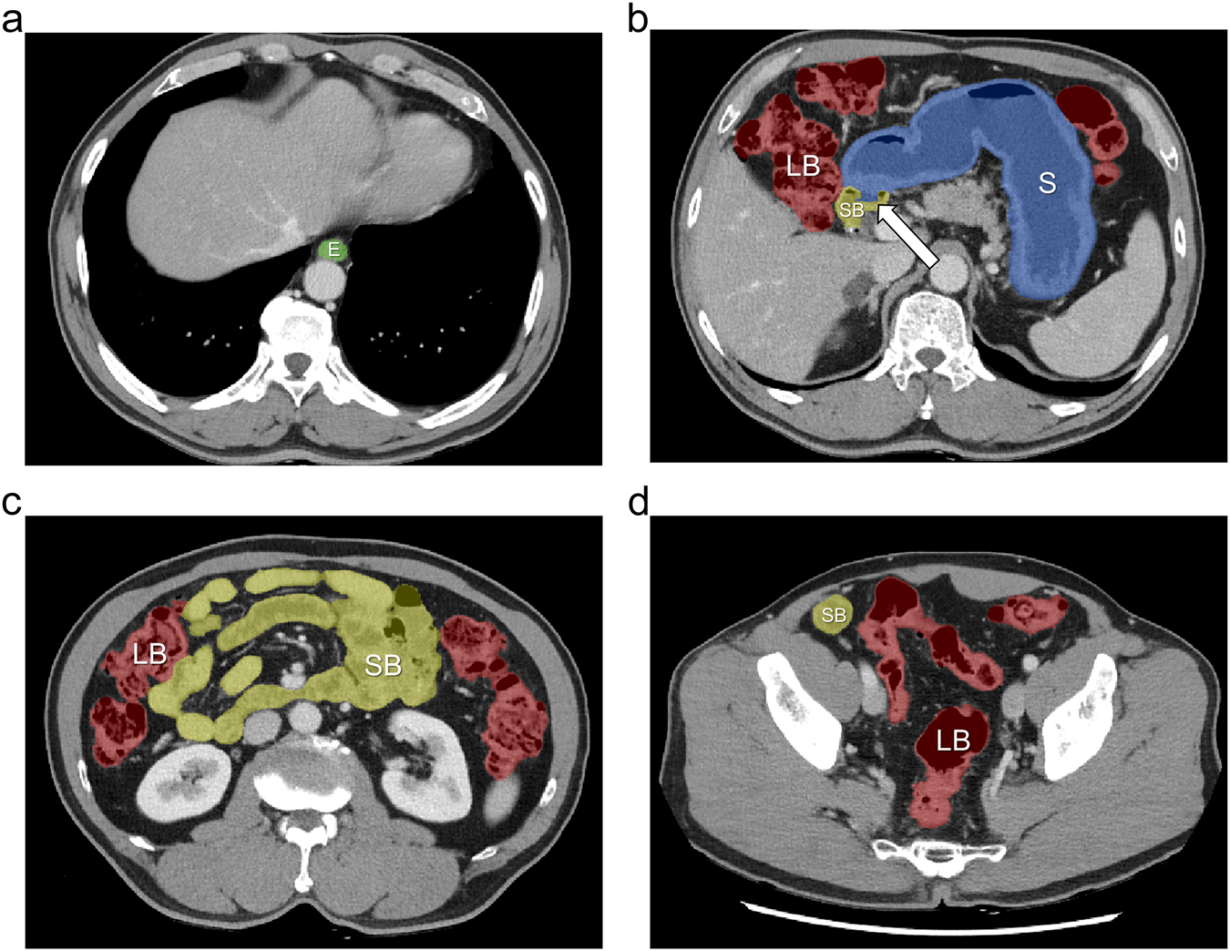
An example of high-fidelity four-part segmentation using our segmentation model. Contrast-enhanced abdominal CT images at the level of the (**a**) lower thorax, (**b**) upper abdomen, (**c**) mid abdomen, and (**d**) pelvis, shown with segmentation masks for the esophagus (E, green), stomach (S, blue), small bowel (SB, yellow), and large bowel (LB, red). Most part of the bowel was correctly segmented, except for a small error at the duodenal bulb, which was incorrectly segmented as part of the stomach (thick arrow). The DSCs for the whole bowel, esophagus, stomach, small bowel, and large bowel were 0.994, 0.951, 0.986, 0.991, and 0.993, respectively

**Fig. 4:**
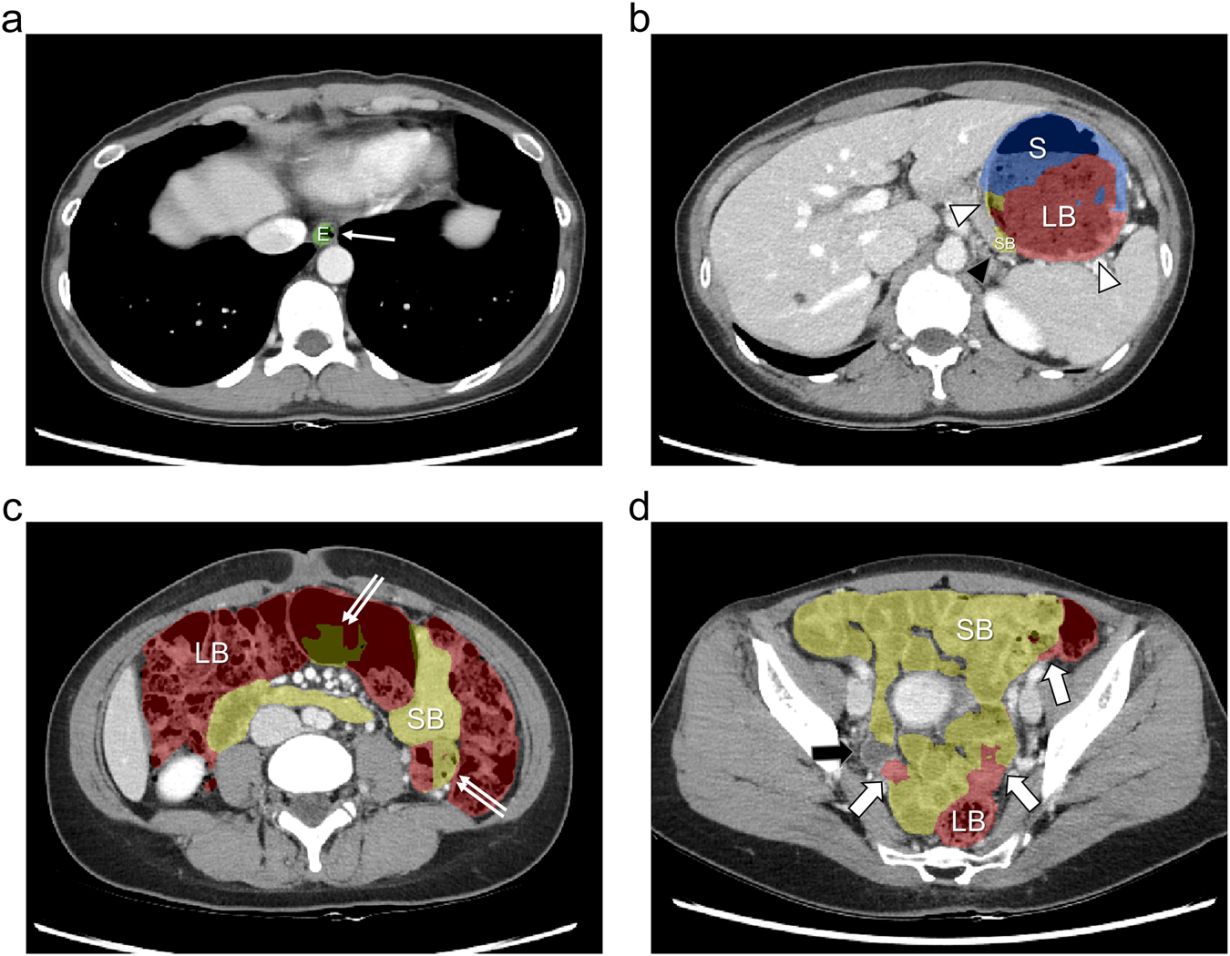
An example of low-fidelity four-part segmentation using our segmentation model. Contrast-enhanced abdominal CT images at the level of the (**a**) lower thorax, (**b**) upper abdomen, (**c**) mid abdomen, and (**d**) pelvis, shown with segmentation masks for the esophagus (E, green), stomach (S, blue), small bowel (SB, yellow), and large bowel (LB, red). **a** A small portion of the esophagus was not segmented (arrow). **b** A substantial portion of the stomach (white arrowheads) and a small portion of the pancreas (black arrowhead) were incorrectly segmented as the large bowel or small bowel. **c** Some portion of the sigmoid colon (double arrows) was incorrectly segmented as the small bowel. **d** A small portion of the small bowel was either not segmented (black thick arrow) or segmented as the large bowel (white thick arrow). The DSCs for the whole bowel, esophagus, stomach, small bowel, and large bowel were 0.964, 0.877, 0.711, 0.850, and 0.851, respectively

Regarding four-part separation, our segmentation model achieved good performance except for the esophagus in the internal dataset. The DSCs for segmentation of the stomach, small bowel, and large bowel were 0.944 ± 0.036, 0.934 ± 0.025, and 0.953 ± 0.018, respectively (Table 2). The model also achieved excellent segmentation accuracy for separation of the stomach, small bowel, and large bowel in the external dataset, with DSCs of 0.970 ± 0.047, 0.960 ± 0.029, and 0.963 ± 0.024, respectively (Table 3). However, the accuracy for esophageal separation was suboptimal in both the internal and external datasets, with DSCs of 0.821 ± 0.044 and 0.807 ± 0.173, respectively. When comparing segmentation performance between the internal and external datasets, the DSC for the stomach (*p* < 0.001) and small bowel (*p* = 0.005) was significantly higher in the external dataset, whereas the DSC for the esophagus (*p* = 0.59) and large bowel (*p* = 0.10) showed no significant difference (Supplementary Table 1). In the external dataset, there were two instances of poor segmentation (DSC < 0.80) for the stomach, none for the small bowel and large bowel, and 18 for the esophagus. For stomach, small bowel, and large bowel segmentation, no significant difference was found in segmentation accuracy in terms of the DSC, sensitivity, and precision between the pre- and post-contrast datasets except for the precision of large bowel segmentation (0.967 ± 0.027 vs 0.958 ± 0.034, *p* = 0.01). For esophageal segmentation, however, the DSC (0.740 ± 0.215 vs 0.874 ± 0.074, *p* < 0.001) and sensitivity (0.681 ± 0.258 vs 0.887 ± 0.116, *p* < 0.001) were significantly lower in the pre-contrast dataset than in the post-contrast dataset (Table 3). Examples of high- and low-fidelity four-part separation performed by our segmentation model are shown in Figs. 3 and 4.

**Fig. 5:**
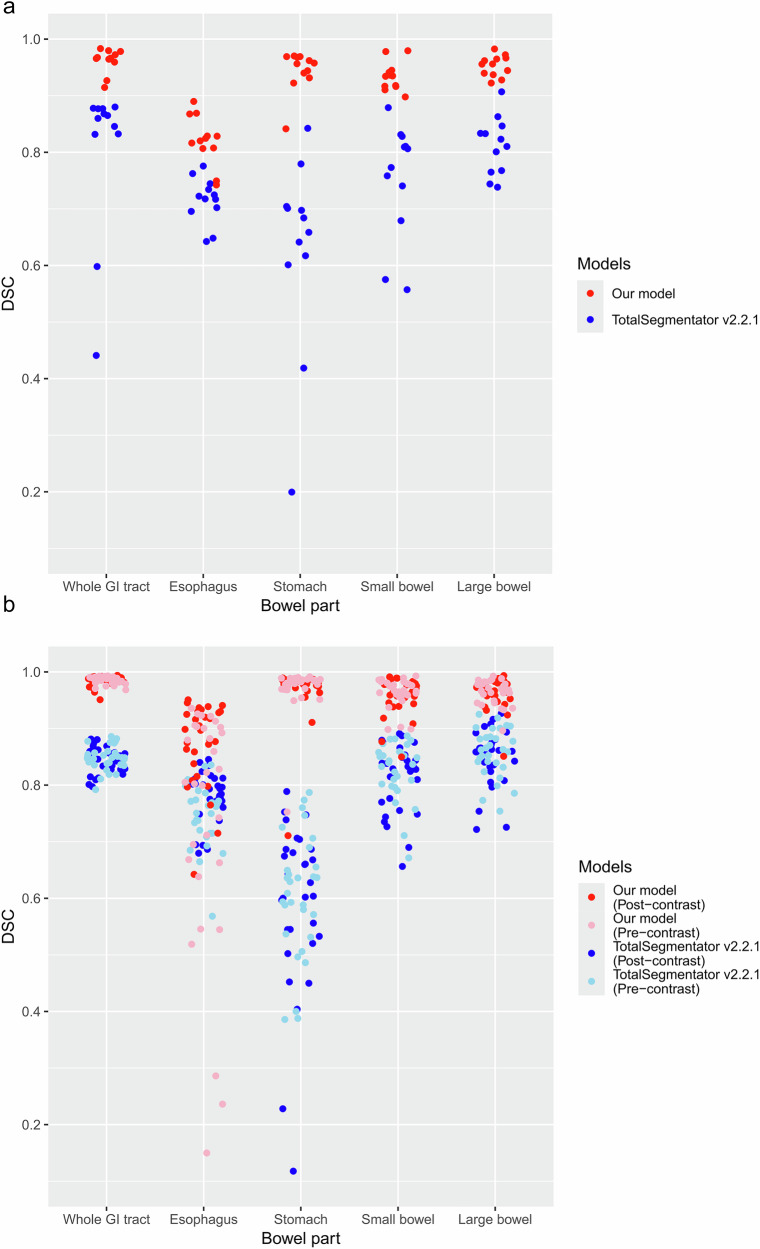
The jitter plots of individual DSCs for (**a**) the internal dataset and (**b**) the external dataset for the whole GI tract and its four subregions, comparing our model (red) and TotalSegmentator v2.2.1 (blue)

When comparing the performance of our model with TotalSegmentator v2.2.1, our model outperformed TotalSegmentator in both the internal and external datasets for whole GI tract segmentation (*p* ≤ 0.001 in terms of DSC). Our model also consistently demonstrated higher DSCs in four-part separation (*p* < 0.001), except for the separation of the esophagus in the external dataset (DSC, 0.807 ± 0.173 vs 0.764 ± 0.052, *p* = 0.07). Additionally, subgroup analyses of pre-contrast (*n* = 30) and post-contrast (*n* = 30) datasets further confirmed our model’s higher DSCs (*p* < 0.001), except for the separation of the esophagus in pre-contrast images (DSC, 0.740 ± 0.215 vs 0.748 ± 0.055, *p* = 0.85) (Tables 2 and 3 and Fig. 5).

### Estimating LBL in patients with and without constipation

There was no significant difference in age between the groups without and with constipation (mean age, 67.6 ± 6.2 vs 66.6 ± 7.3 years on a per-exam basis, *p* = 0.64). However, the proportion of women was significantly higher in the constipation group (22.0% vs 54.2%, *p* = 0.003). Additionally, there were significant differences in height (165.7 ± 9.3 vs 161.7 ± 7.6 cm, *p* = 0.02) and the CT protocol (*p* = 0.03) between the two groups (Table 1).

**Fig. 6:**
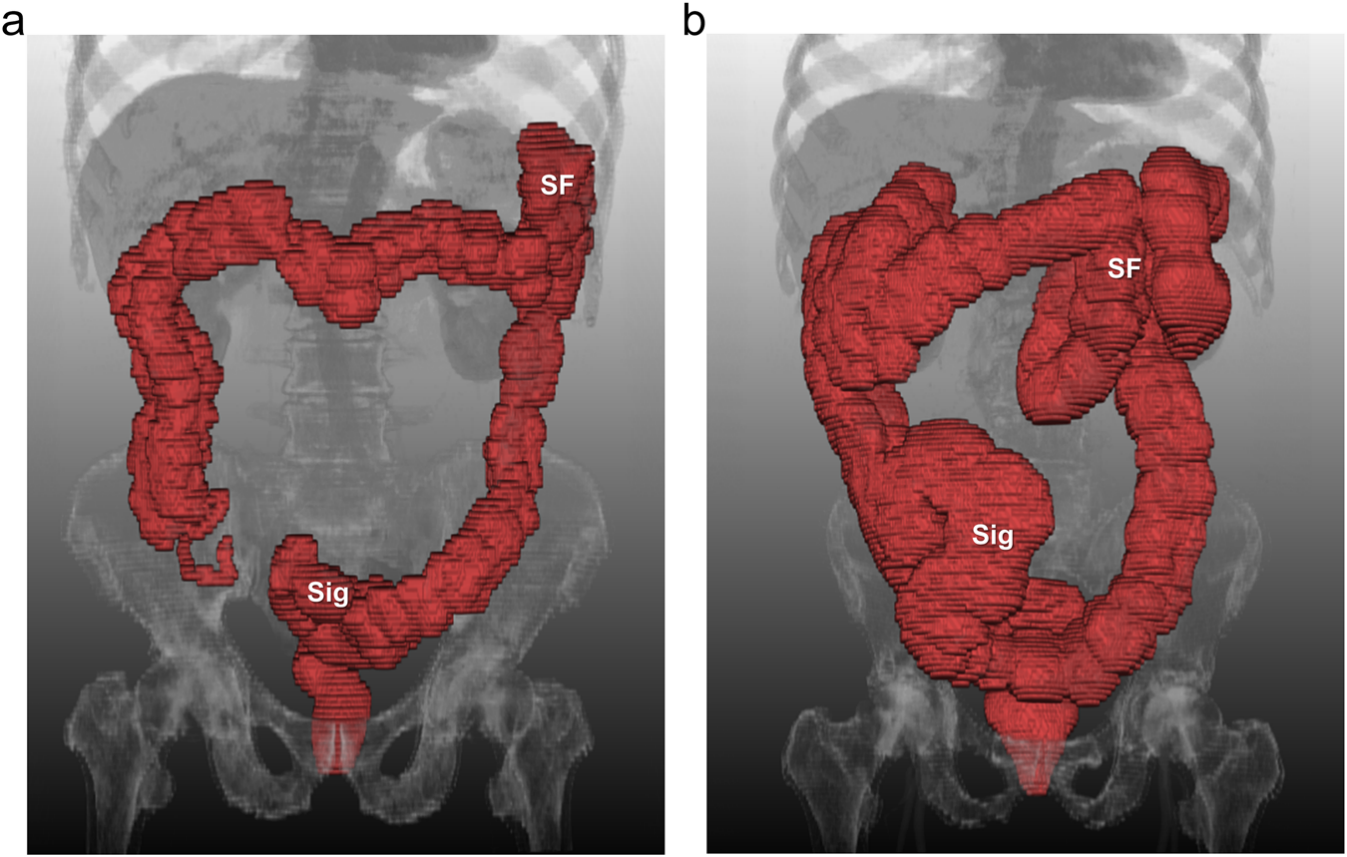
Examples of automatically segmented colon masks in 3D reconstruction images and LBL measurements for (**a**) a 70-year-old male patient (height, 1.57 m) who did not have constipation and (**b**) another 73-year-old male patient (height, 1.67 m) who had constipation. The patient with constipation had a longer large bowel with a more complex course, especially at the splenic flexure (SF) and sigmoid colon (Sig). The LBL corrected for height (64.5 cm/m vs 107.7 cm/m) and height squared (41.1 cm/m^2^ vs 64.5 cm/m^2^) is longer in the patient with constipation

The mean LBL was significantly longer in the group with constipation on a per-exam basis (131.1 ± 21.2 vs 143.1 ± 24.1 cm, *p* = 0.01), but statistical significance was not achieved on a per-subject basis (128.3 ± 23.0 vs 140.3 ± 26.1 cm, *p* = 0.10). However, when adjusting for differences in LBL by dividing LBL by height or height squared, both mean LBL/height and mean LBL/height^2^ exhibited significantly higher values in the group with constipation in both per-subject and per-exam comparisons (mean LBL/height, 77.6 ± 13.6 vs 86.9 ± 17.1 cm/m on a per-subject basis, *p* = 0.04, and 79.1 ± 12.4 vs 88.8 ± 15.8 cm/m on a per-exam basis, *p* = 0.001; mean LBL/height^2^, 47.1 ± 8.6 vs 53.9 ± 11.8 cm/m^2^ on a per-subject basis, *p* = 0.02, and 47.9 ± 7.9 vs 55.2 ± 10.9 cm/m^2^ on a per-exam basis, *p* < 0.001) (Table 4, Fig. 6, and Supplementary Fig. 2).

**Table 4:** Comparison of LBL between patients with and without constipation

	Per-subject basis^a^			Per-exam basis		
	Normal bowel habit (*n* = 22)	Constipation (*n* = 29)	*p*-value	Normal bowel habit (*n* = 41)	Constipation (*n* = 59)	*p*-value
LBL (cm)	128.3 ± 23.0	140.3 ± 26.1	0.10	131.1 ± 21.2	143.1 ± 24.1	0.01
LBL/height (cm/m)	77.6 ± 13.6	86.9 ± 17.1	0.04	79.1 ± 12.4	88.8 ± 15.8	0.001
LBL/height^2^ (cm/m^2^)	47.1 ± 8.6	53.9 ± 11.8	0.02	47.9 ± 7.9	55.2 ± 10.9	< 0.001

Data are presented with mean ± standard deviation

*LBL* large bowel length

^a^ For subjects who underwent more than one CT examination, the latest CT scan was used for the analysis

In the subgroup where human-generated LBL was also calculated, the intraclass correlation coefficient between model-calculated and human-generated LBL was 0.994 (95% confidence interval, 0.977–0.998), demonstrating excellent agreement. However, manually segmenting the large bowel to calculate human-generated LBL took an average of 24.9 ± 11.1 min per case. In this subgroup, both model-calculated and human-generated LBL showed a trend of being longer in the constipation group when corrected for height or height squared, though the difference did not reach statistical significance (*p* = 0.09–0.18) (Supplementary Table 3).

## Discussion

This study successfully developed and validated a two-step deep neural network for whole GI tract segmentation and four-part separation from CT scans. We adopted a two-step segmentation approach to ensure stepwise optimization for accuracy, manage computational constraints, and improve adaptability for various applications, enabling the use of both whole GI tract and subregion segmentations. Our model achieved high DSC, sensitivity, and precision, all above 0.98 in the external dataset obtained from a different hospital for whole GI tract segmentation. This performance surpasses the models in other studies, which reported DSC scores ranging from 0.75 to 0.95 for segmenting whole or part of the GI tract using abdominal CT or MRI data [15, 19, 23–26]. Moreover, our model also demonstrated high DSCs for the separation of the stomach, small bowel, and large bowel, exceeding 0.95 in the external dataset. The only exception was the esophagus, which showed slightly diminished segmentation accuracy, with most cases of poor segmentation (DSC < 0.80) occurring particularly in pre-contrast images. This may be partly due to its small size and close proximity to adjacent structures, such as the aorta, left atrium, and azygos vein, making differentiation more challenging. In addition, our model showed superior performance in whole bowel segmentation and four-part separation, except for the separation of the esophagus, compared to TotalSegmentator v2.2.1 in both the pre- and post-contrast CT scans. This suggests that our model maintains robust performance even in the absence of contrast agent administration.

The segmentation performance was better in the external dataset compared to the internal dataset, despite using data from a different hospital for the external dataset. This result may be attributed to two factors: first, the small sample size used for internal testing might have limited the robustness of the evaluation, and second, unlike the internal dataset, which consisted of CT scans with various protocols, the external dataset was composed exclusively of abdominal CT scans, which may have been relatively easier for segmentation. Further validation using larger and more diverse datasets would help to confirm our findings.

In a clinical application, we compared LBL between patients with and without constipation from another hospital, which was distinct from both the training and external test datasets. Our findings revealed a significant association between constipation and increased LBL, adjusted for height differences. These results suggest that constipation influences the anatomical characteristics of the large bowel; however, causality remains indeterminate, considering a previous report indicating that constipation may contribute to an increase in colon length [27]. Our findings align with a recent study reporting that LBL was associated with bowel habits and sex, and specifically that longer LBL tended to be associated with less frequent defecation [9]. Furthermore, another study using MRI data to compare LBL between pediatric patients with and without constipation found longer LBL in those with constipation [28]. However, those studies relied on manual segmentation of the large bowel, a process that is time- and labor-consuming and challenging to implement in real clinical settings due to its resource demands. In contrast, our model can accurately segment the colon from CT images of the GI tract, demonstrating the potential for a clinically feasible application in practice. Nevertheless, the lack of true reference values (gold-standard measurements obtained in a surgical setting) limits its immediate clinical applicability, despite the high agreement between AI-assisted segmentation and radiologists. Furthermore, as our study was based on a limited sample size, further validation with larger, prospectively collected datasets is needed.

No standard method exists for accurately measuring the length of the small intestine, but using a silk suture or umbilical tape directly in the operating room is considered to be relatively reliable [29]. Although there has been a successful attempt to measure the length of the small bowel using MR enterography in a recent paper [11], manual measurement of the small bowel on conventional CT or MR is challenging due to the difficulty of tracing this elongated organ. Precisely measuring the length of the small bowel on CT images could significantly benefit patients with Crohn’s disease who require surgery by assisting in surgical planning and postoperative care strategies to prevent short bowel syndrome. Our model has the capability to selectively segment the small bowel in both pre- and post-contrast CT scans. This implies that quantifying other characteristics of the diseased bowel beyond length may be feasible; possible directions for further research could include the degree of contrast enhancement or iodine concentration, which reflects the degree of inflammation [30]. The small bowel is longer and has a more complex course than the large bowel, and is mostly collapsed on routine abdominal CT. Therefore, even with accurate segmentation, it would be difficult to measure the exact length using a method similar to our colon length measurements. Nonetheless, if our model can be applied to CT enterography in further studies, it might be possible to measure the small bowel length through segmentation, which could provide clinical benefits for patients with small bowel diseases such as Crohn’s disease.

Our study has some limitations. First, while the deep learning model developed in the study demonstrated high accuracy with our datasets, they were obtained from patients without a history of GI tract surgery and mostly without GI disease. Especially, the external validation was conducted exclusively with abdominal CT scans, highlighting the need for further validation with more diverse datasets. The generalizability our model to datasets with various diseases and regions needs further validation. Second, given the suboptimal accuracy of esophageal segmentation, the model needs to be improved to increase segmentation accuracy for this region. Third, the ground-truth masks were generated semiautomatically by using the automatically generated internal organ masks. Although the radiologists carefully reviewed and corrected the masks slice-by-slice, the final ground-truth masks may still have been influenced to some extent by the initially generated internal organ masks, leading to potential automation bias. Fourth, our estimation of LBL did not account for the actual anatomy of the large bowel, such as non-round cross-sectional areas and periodic luminal changes due to haustra. We initially attempted to measure LBL using a mathematical algorithm, such as the Floyd-Warshall method, for the 3D volume mask of the large bowel. However, this approach was particularly challenging in cases where parts of the large bowel abutted other bowel segments, hindering the identification of the bowel trajectory with the algorithm. Fifth, the estimated LBL was not validated against a reference standard measurement, such as a surgeon’s manual measurement of colon length using a ruler during an operation. Nevertheless, there is currently a lack of tools to measure bowel length noninvasively, which highlights the value of our approach for estimating LBL.

In conclusion, our study successfully developed a deep learning model for accurate GI tract segmentation from CT scans and demonstrated the valuable clinical implications of this segmentation model by noninvasively measuring bowel length. Further refinement of segmentation algorithms and larger-scale clinical studies are warranted to enhance the clinical utility of our findings and facilitate advancements in disease monitoring and patient care in GI diseases.

## Supplementary information

ELECTRONIC SUPPLEMENTARY MATERIAL

## Abbreviations

DSC: Dice similarity coefficient
GI: Gastrointestinal
LBL: Large bowel length
VNC: Virtual non-contrast CT
WBCT: Whole-body CT
