# Supplementary material for: Deep learning for automatic volumetric bowel segmentation on body CT images
Source: Eur Radiol. 2025 May 2;35(11):7307–19. doi: 10.1007/s00330-025-11623-z (PMC12559117; doi:10.1007/s00330-025-11623-z)
Supplement: Supplementary file 1 — ELECTRONIC SUPPLEMENTARY MATERIAL [file 330_2025_11623_MOESM1_ESM.pdf]

Deep Learning for Automatic Volumetric Bowel Segmentation on Body CT Images

ELECTRONIC SUPPLEMENTARY MATERIAL

CT acquisition for the development dataset

Our developmental dataset included 1) contrast-enhanced abdominal CT with or without paired virtual non-contrast CT (VNC) images, 2) contrast-enhanced chest CT with paired VNC images, and 3) non-enhanced whole-body CT (WBCT) as part of whole-body PET-CT scans. 1) For contrast-enhanced abdominal CT, we used scans from asymptomatic adult outpatients who did not have any focal lesions in the abdomen except small cysts in the the liver, spleen, kidney or pancreas, which were performed using two different dual-energy CT machines: SOMATOM Force (Siemens Healthineers) or IQon Spectral CT (Philips Healthcare). When clinically obtained VNC images were available, paired VNC images were also included in the study (1). 2) For contrast-enhanced chest CT, we used virtual 0.7-mm-section 50-keV contrast-enhanced images and VNC images from dual-energy pulmonary CT angiograms obtained using the Somatom Force (Siemens Healthineers) (2). 3) For WBCT, we used non-contrast CT images from PET/CT scans performed for the initial staging workup of early lung cancer or systematic screening, acquired with the Biograph40, mBiograph64 (Siemens Healthineers), or GEMINI (Philips Healthcare) systems (3).

Definition of the dice similarity coefficient, sensitivity, and precision

The dice similarity coefficient (DSC) provides the similarity between ground-truth and network-derived masks by measuring the ratio between the number of voxels included in both masks (i.e., true-positive voxels) and the average number of voxels in ground-truth and network-derived masks. Sensitivity is defined as the ratio between the number of true-positive voxels and voxels in ground-truth masks, and precision is defined as the ratio between the number of true-positive voxels and voxels in network-derived masks.

Comparison between our model and TotalSegmentator v2.2.1.

TotalSegmentator v2.2.1 divided the GI tract into the following structures: esophagus, stomach, duodenum, small bowel, and colon, whereas our model did not distinguish between the duodenum and small bowel (4). Therefore, for a comparison between our model and TotalSegmentator v2.2.1, we redefined the small bowel mask by combining the duodenum and small bowel masks segmented by TotalSegmentator v2.2.1. The whole GI tract mask for TotalSegmentator v2.2.1 was defined by merging the esophagus, stomach, duodenum, small bowel, and colon masks.

References

1. Jeon SK, Joo I, Park J, Kim J-M, Park SJ, Yoon SH. Fully-automated multi-organ segmentation tool applicable to both non-contrast and post-contrast abdominal CT: deep learning algorithm developed using dual-energy CT images. Sci Rep 2024;14(1):4378. DOI: 10.1038/s41598-024-55137-y

2. Nam JG, Witanto JN, Park SJ, Yoo SJ, Goo JM, Yoon SH. Automatic pulmonary vessel segmentation on noncontrast chest CT: deep learning algorithm developed using spatiotemporally matched virtual noncontrast images and low-keV contrast-enhanced vessel maps. Eur Radiol 2021;31(12):9012-9021. DOI: 10.1007/s00330-021-08036-z

3. Lee YS, Hong N, Witanto JN, Choi YR, Park J, Decazes P, Eude F, Kim CO, Kim HC, Goo JM. Deep neural network for automatic volumetric segmentation of whole-body CT images for body composition assessment. Clin Nutr 2021;40(8):5038-5046. DOI: 10.1016/j.clnu.2021.06.025

4. Wasserthal J, Breit H-C, Meyer MT, Pradella M, Hinck D, Sauter AW, Heye T, Boll DT, Cyriac J, Yang S. Totalsegmentator: Robust segmentation of 104 anatomic structures in ct images. Radiol Artif Intell 2023;5(5):e230024. DOI: 10.1148/ryai.230024

Supplementary Figure 1. The jitter plots of individual Dice Similarity Coefficients (DSCs) of the preliminary masks created by MEDIP software for the whole gastrointestinal tract segmentation.

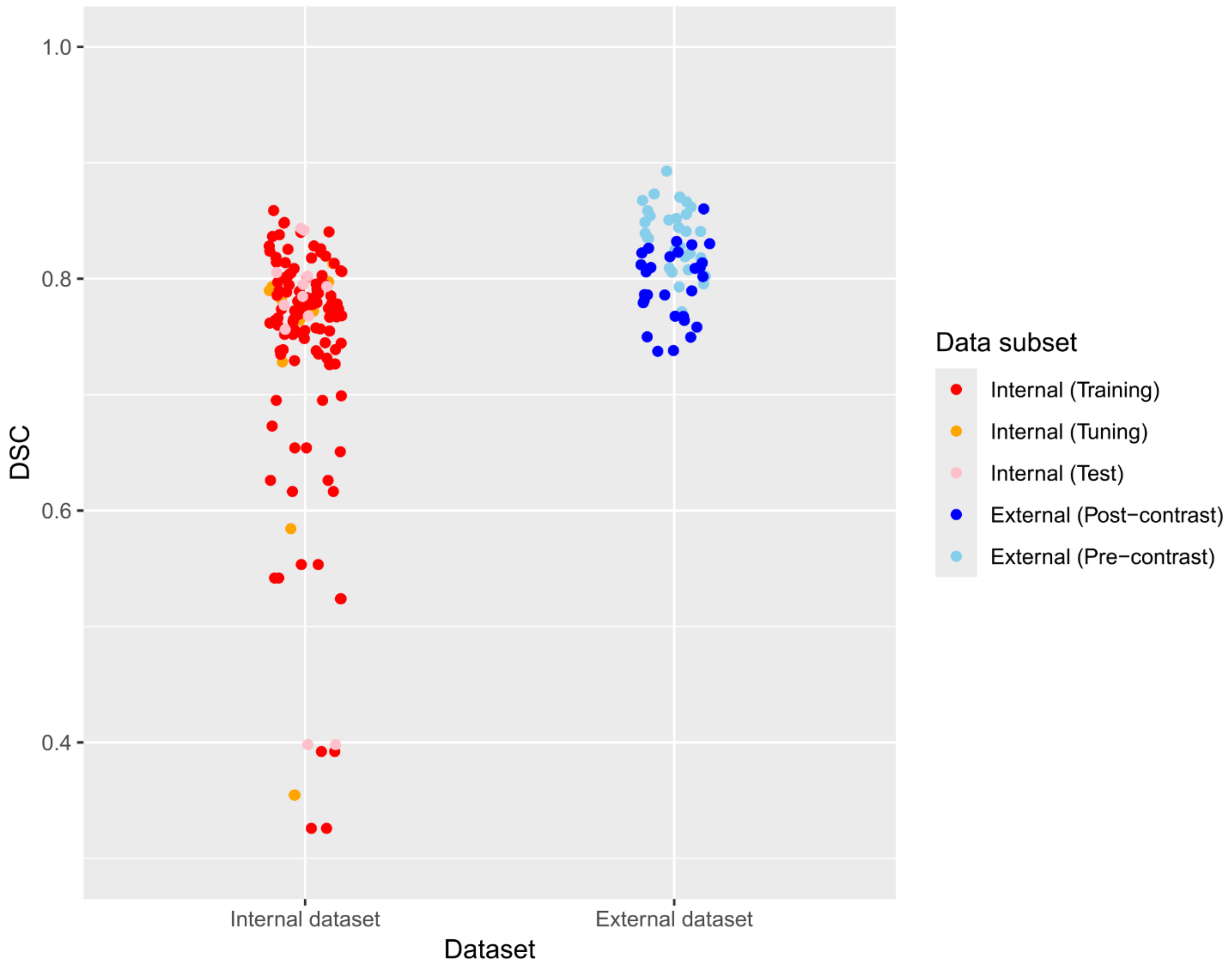

Supplementary Figure 2. Example of changes in large bowel length (LBL) over time in a 64-year-old (at initial CT) female patient with constipation. (a) Automated large bowel segmentation (red) on initial CT. LBL measured 165.9 cm. (b) Automated large bowel segmentation (red) on follow-up CT after 5 years. LBL increased to 181.0 cm, with a more tortuous large bowel course. (c) Automated large bowel segmentation (red) on follow-up CT after 10 years. LBL further increased to 195.8 cm, and the large bowel became even more tortuous and dilated.

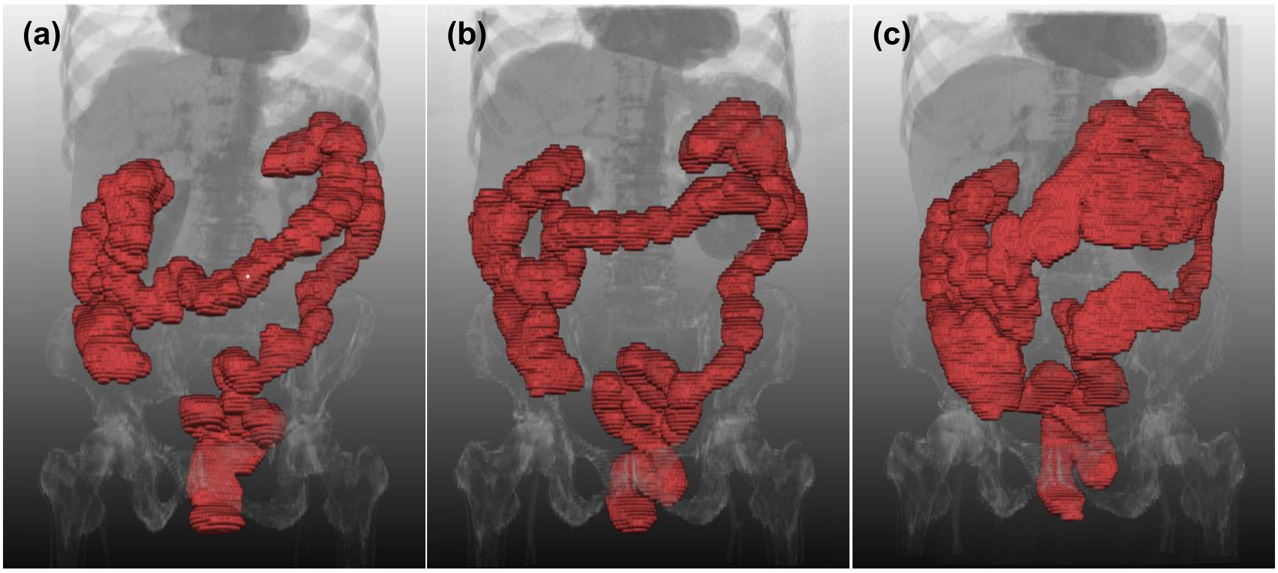

Supplementary Table 1. Comparison of the segmentation performance between the internal and external datasets

	Internal dataset			External dataset			p-value		
	DSC	Sensitivity	Precision	DSC	Sensitivity	Precision	DSC	Sensitivity	Precision
Whole GI tract segmentation	0.962 ± 0.021	0.970 ± 0.011	0.954 ± 0.033	0.985 ± 0.008	0.985 ± 0.011	0.984 ± 0.007	< 0.001	0.001	< 0.001
Four-part separation									
- Esophagus	0.821 ± 0.044	0.829 ± 0.063	0.826 ± 0.099	0.807 ± 0.173	0.784 ± 0.224	0.877 ± 0.066	0.59	0.20	0.11
- Stomach	0.944 ± 0.036	0.962 ± 0.016	0.929 ± 0.061	0.970 ± 0.047	0.968 ± 0.074	0.975 ± 0.020	< 0.001	0.55	< 0.001
- Small bowel	0.934 ± 0.025	0.931 ± 0.034	0.938 ± 0.024	0.960 ± 0.029	0.963 ± 0.031	0.958 ± 0.035	0.005	0.009	0.007
- Large bowel	0.953 ± 0.018	0.963 ± 0.016	0.943 ± 0.030	0.963 ± 0.024	0.964 ± 0.028	0.962 ± 0.031	0.10	0.75	0.06

Note - data are presented with mean ± standard deviation

DSC, dice similarity coefficient; GI, gastrointestinal

Supplementary Table 2. The performance of our model and preliminary mask created by MEDIP software for segmentation of the gastrointestinal tract

	Our model			Preliminary mask			P-value		
	DSC	Sensitivity	Precision	DSC	Sensitivity	Precision	DSC	Sensitivity	Precision
Internal dataset (n = 12)	0.962 ± 0.021	0.970 ± 0.011	0.954 ± 0.033	0.730 ± 0.157	0.973 ± 0.017	0.605 ± 0.170	< 0.001	0.62	< 0.001
External dataset									
Whole dataset (n = 60)	0.985 ± 0.008	0.985 ± 0.011	0.984 ± 0.007	0.816 ± 0.036	0.996 ± 0.007	0.693 ± 0.052	< 0.001	< 0.001	< 0.001
Pre-contrast (n = 30)	0.986 ± 0.007	0.985 ± 0.010	0.986 ± 0.006	0.836 ± 0.028	0.994 ± 0.009	0.723 ± 0.042	< 0.001	< 0.001	< 0.001
Post-contrast (n = 30)	0.984 ± 0.009	0.986 ± 0.012	0.983 ± 0.007	0.795 ± 0.031	0.998 ± 0.002	0.662 ± 0.043	< 0.001	< 0.001	< 0.001

Note - data are presented with mean ± standard deviation

DSC, dice similarity coefficient; GI, gastrointestinal

Supplementary Table 3. Comparison of model-calculated and human-generated large bowel length between patients with and without constipation

	Model-calculated			Human-generated		
	**Normal bowel habit (n = 10)**	**Constipation (n = 10)**	**p-value**	**Normal bowel habit (n = 10)**	**Constipation (n = 10)**	**p-value**
LBL (cm)	126.7 ± 21.0	138.1 ± 22.2	0.26	126.2 ± 20.1	136.0 ± 22.8	0.33
LBL/height (cm/m)	77.1 ± 13.2	86.9 ± 14.8	0.14	76.8 ± 12.8	85.6 ± 15.2	0.18
LBL/height2 (cm/m2)	47.0 ± 8.9	54.7 ± 10.1	0.09	46.9 ± 8.7	53.9 ± 10.3	0.12

Note - data are presented with mean ± standard deviation

LBL, large bowel length
